# *Meloidogyne aegracyperi* n. sp. (Nematoda: Meloidogynidae), a root-knot nematode parasitizing yellow and purple nutsedge in New Mexico

**DOI:** 10.21307/jofnem-2019-071

**Published:** 2019-10-25

**Authors:** J. D. Eisenback, L. A. Holland, J. Schroeder, S. H. Thomas, J. M. Beacham, S. F. Hanson, V. S. Paes-Takahashi, P. Vieira

**Affiliations:** 1 School of Plant and Environmental Science, Virginia Tech, Blacksburg, VA, 24061; 2 Department of Plant Pathology, University of California-Davis, Davis, CA, 95616; 3 Department of Entomology, Plant Pathology and Weed Science, New Mexico State University, Las Cruces, NM, 88003; 4 Department of Plant Protection, Universidade Estadual Paulista “Julio Mesquita Filho” (UNESP/FCAV), Jaboticabal, SP, 14884900

**Keywords:** *Cyperus*, *C. esculentus*, *C. rotundus*, Description, Host range, Morphology, Morphometrics, Scanning electron microscopy, Taxonomy

## Abstract

*Meloidogyne aegracyperi* n. sp. is described from roots of purple nutsedge in southern New Mexico, USA. Mature females are small (310–460 µm), pearly white, with their egg masses completely contained inside root galls. The neck is often at a 90 to 130° angle to the protruding posterior end with the perineal pattern. The distance of the dorsal esophageal gland orifice (DGO) to the base of the stylet is relatively long (4.0–6.1 µm), and the excretory pore is level with the base of the stylet. The anterior portion of the rounded lumen lining of the metacorpus contains 3 to 10 small vesicles. The perineal pattern has a rounded dorsal arch with a tail terminal area that is smooth or marked with rope-like striae. Only two males were found. The body twists 90° throughout its length. The DGO to the base of the stylet is long (3.0–3.3) µm. The cephalic framework of the second-stage juvenile is weak, and the stylet is short (10.1–11.8 µm). The DGO to the base of the stylet is long (3–5 µm). The tail is very long (64–89 µm) and the hyaline portion of the tail is very narrow, making the tail finely pointed. Eggs are typical for the genus and vary in length (85.2–99.8 µm) and width (37.1–48.1 µm), having a L/W ratio of (2.1–2.6). Maximum likelihood phylogenetic analyses of the different molecular loci (partial 18S rRNA, D2-D3 of 28S rRNA, internal transcribed spacer (ITS) rRNA, cytochrome oxidase subunit II (COII)-16S rRNA of mitochondrial DNA gene fragments and partial *Hsp90* gene) placed this nematode on an independent branch in between *M. graminicola* and *M. naasi* and a cluster of species containing *M. chitwoodi*. *M. fallax*, and *M. minor*. Greenhouse tests showed that yellow and purple nutsedge were the best hosts, but perennial ryegrass, wheat, bentgrass, and barley were also hosts.

Yellow nutsedge (*Cyperus esculentus* L.) and purple nutsedge (*C. rotundus* L.) are perennial weeds of global importance that can enhance survival and population densities of *Meloidogyne incognita* (Kofoid and White, 1919) Chitwood, 1949 and result in injury to crops (Schroeder et al., 1993, 1994, 2004; Thomas et al., 2004). Shoot growth of both nutsedges is not affected by *M. incognita* parasitism; numbers and size of reproductive tubers increase as nutrient resources are reallocated to roots, and galls do not develop on roots (Mauk et al., 1999; Schroeder et al., 1999). During routine bioassays of nutsedge cultures maintained in the greenhouse, galling was observed on roots of purple nutsedge, but was not present on any comingled roots of *M. incognita*-susceptible ‘Rutgers’ tomato (*Solanum lycopersicum* L.). Subsequent dissection of the galls revealed small, mature *Meloidogyne* females and egg masses that were primarily contained inside the root tissue. Eggs were recovered from roots of purple nutsedge, but not that of tomato following extraction with sodium hypochlorite. Additional pots of purple nutsedge and Rutgers tomato that were established in pasteurized soil using surface-sterilized nutsedge tubers and inoculated with eggs recovered from galled purple nutsedge roots showed similar results: root galling and egg recovery from nutsedge, but no galling or egg recovery from tomato.

Additional research on the morphology and host range conducted at Virginia Tech and New Mexico State University revealed several unusual morphological characters and a unique host range that indicated it was a new species. The perineal pattern, shape of the female stylet, and shape of the male head and stylet were unique and different from those of any other described species. *Meloidogyne aegracyperi* n. sp. is described herein, and the common name ‘nutsedge root-knot nematode’ is proposed. The specific epithet was derived from the Latin word ‘aegra’ and the host plant name, meaning ‘diseased *Cyperus*.’

## Materials and methods

*Meloidogyne aegracyperi* n. sp. was established from field collected soil and plants from the type locality and propagated on purple nutsedge to maintain stock cultures that were kept in a greenhouse at 22 to 28°C. All nematodes used in morphologic, morphometric, and host range studies were from these cultures.

## Morphological studies

Males and second-stage juveniles (J2) were extracted from washed galled roots incubated in a moist chamber. Light microscopy (LM) observations were made from specimens mounted on 5% water agar pads and paralyzed with 0.1 m sodium azide (Eisenback, 2012). Females and J2 were prepared for scanning electron microscopy (SEM) according to Eisenback (1985). Perineal patterns were prepared for SEM according to Charchar and Eisenback (2001). They were observed and photographed by LM with negative contrast as reported by Eisenback (2012). Eggs were measured in fresh tap water mounted on agar pads. All LM observations except for perineal patterns were made with a bright field microscope, and at least 100 specimens were observed. However, only two males were recovered from hundreds of infected nutsedge plants. Measurements were made of females in 2% glutaraldehyde in 0.1 M cacodylic acid buffer, pH 7.2; perineal patterns were mounted in glycerin, and extracted stylets (Eisenback et al., 1980) were measured with the SEM. In total, 30 specimens of J2 and females were randomly selected for measurements along with the two males.

## Host range test

Seedlings of alfalfa (*Medicago sativa* L.) cv. Doña Ana, chile pepper (*Capsicum annuum* L.) cv. NM 6-4, corn (*Zea mays* L.) DynaGro 58UP30, cotton (*Gossypium hirsutum* L.) cv. Deltapine 393, onion (*Allium cepa* L.) cv. nuMex Dulce, sorghum (*Sorghum bicolor* (L.) Moench.) cv. Sordan Headless, common oats (*Avena sativa* L.), winter rye (*Secale cereale* L.), perennial ryegrass (*Lolium perenne* L.) cv. Barlennium, wheat (*Triticum aestivum* L.), barley (*Hordeum vulgare* L.) cv. Robust, tomato (*Solanum lycopersicum* L.) cv. Rutgers, purple nutsedge (*Cyperus rotundus* L.), yellow nutsedge (*Cyperus esculentus* L.) were transplanted as single plants into 11 cm diameter clay pots containing 500 cm^3^ of sterilized sandy loam soil. They were inoculated with a suspension of 3,000 freshly hatched juveniles in 50 ml of water in holes around the root system. Each treatment was replicated 10 times and the plants were kept in a greenhouse at 22 to 28°C. Roots were washed with tap water and stained with phloxine B (Dickson and Struble, 1965) to aid in the counting of egg masses.

## Molecular studies

### Sample isolation

In total, 24 individual females were isolated from nutsedge roots and transferred into separate polymerase chain reaction (PCR) tubes containing 20 μl of lysis buffer (0.2 M Tris-Cl, pH 7.8) and stored at −20°C.

### Lysis, PCR, and sequencing

Lysis was performed using a rapid single tube lysis procedure (Solano and Hanson, unpubl. data). Briefly, samples were removed from −20°C and incubated at 90°C for 10 min. After heating, 30 μl of Proteinase K digestion mixture was added to each sample (5 μl Proteinase K (Qiagen Inc., Valencia, CA), 3 μl of 10× Platinum Taq DNA Polymerase PCR Buffer (Invitrogen, Carlsbad CA), and 22 μl water per sample). Samples were then sonicated for 8 min in a Branson 2510 ultrasonic cleaner then incubated for 30 min at 60°C. After Proteinase K digestion samples were frozen at −80°C for 10 min then incubated at 90°C for 10 min. After heating, 50 μl of water was added to each tube and samples were well mixed then stored at −20°C prior to PCR.

Sequence-based identification of individual nematodes was performed using 18S rRNA and heat shock protein 90 (*Hsp90*) markers with primers used for amplification and sequencing listed in Table 1. Amplification of the 18S rRNA was performed using two primer sets; 79 F deg plus 1629 R deg which amplifies the majority of the 18S rRNA gene and 983 F deg plus Nema 28S R AG which amplifies the 3′ ~1/2 of the 18S rRNA gene and the ITS region. The previously described primer set RKN-d1F plus RKN-5R was used for amplification and sequencing of the *Hsp90* gene (Skantar and Carta, 2004). All amplification reactions were performed in 40 μl PCR reactions using NEBNext Q5 Hot Start HiFi PCR Master Mix (New England BioLabs, Ipswich, MA). PCR reactions contained: 2 μl of nematode lysate, 4 μl of each primer, 17.2 μl of water, and 20 μl of the 2× PCR master mix. The cycling conditions for the 18S rRNA reactions were 94°C for 2 min, followed by 34 cycles of 94°C for 20 sec, 58°C for 30 sec, and 65°C for 80 sec, with a final extension at 68°C for 10 min. Cycling conditions used for the *Hsp90* gene were 94°C for 2 min, followed by 40 cycles of 94°C for 30 sec, 55°C for 20 sec, and 68°C for 90 sec, with a final extension at 68°C for 5 min. PCR products were run on a 1% agarose gel in SB buffer (Brody and Kern, 2004) and stained with SYBR Gold according to manufacturer’s instructions (Invitrogen Inc., Carlsbad, CA). Gels were visualized on a digital gel imager (UVP EC3 Imaging System, UVP Inc). PCR reactions that contained the expected size amplicons were treated with ExoSAP-IT (Affymatrix Inc., Santa Clara, CA) according to manufacturer’s instructions. Amplicon concentrations in ExoSAP-IT-treated reactions were determined using a commercial SYBR green-I based DNA quantification kit (Invitrogen Inc., Carlsbad, CA) and read on a fluorescent plate reader (Synergy HTX Multi-Mode Microplate Reader). Automated dideoxy sequencing was performed by Genewiz Inc. (South Plainfield, NJ). Sequence editing, assembly, and analysis were performed using the integrated sequence analysis package, Genious 9.0.2 (Kearse et al., 2012) with the MAFFT aligner being used to generate multiple sequence alignments. Maximum likelihood phylogenetic trees were generated using Mega 6.06 with default parameters and 500× bootstrapping (Tamura et al., 2013).

**Table 1.** Primers used to compare *M. aegracyperi* n. sp. with its closest relatives.

Primer	Sequence (5′-3′)	Use	Marker
D2A	ACAAGTACCGTGAGGGAAAGTTG	PCR and sequencing	18S rRNA
D2B	TCGGAAGGAACCAGCTACTA	PCR and sequencing	18S rRNA
ITS1	CGTAACAAGGTAGCTGTAG	PCR and sequencing	ITS rRNA
ITS2	TTTCACTCGCCGTTACTAAGG	PCR and sequencing	ITS rRNA
1618 F	TTT GTA CAC AC GCC CGT CG	Sequencing	18S rRNA
1421 F deg	GGT CTG TGA TGC CCT WRG ATG T	Sequencing	18S rRNA
546 F	GGG CAA GTC TGG TGC CAG CAG	Sequencing	18S rRNA
Nema 28 S R AG	ACT CCT TGG TCC GTG TTT CAA GA	PCR	18S rRNA
983 F deg	CGA MRG YGA TYA GAT ACC GCY	PCR	18S rRNA
1629 R deg	GGT GTG TAC AAA KSR CAG GGA	PCR	18S rRNA
79 F deg	GDG AAACYG CGWACG GCT	PCR	18S rRNA
RKN-5R	TCG AAC ATG TCA AAA GGA GC	PCR and sequencing	HSP90
RKN-d1F	GCY GAT CTT GTY AAC AAC CYT GGA AC	PCR and sequencing	HSP90

For the amplification of the D2-D3 region of the 28S rRNA, the forward D2A (5′-ACAAGTACCGTGAGGGAAAGTTG-3′) and the reverse D3B (5′-TCGGAAGGAACCAGCTACTA-3′) primers were used (De Ley et al., 1999). For amplification of the ITS1/ITS2 region of the rRNA, the forward primer 5′-CGTAACAAGGTAGCTGTAG-3′ (Ferris et al., 1993) and reverse primer 5′-TTTCACTCGCCGTTACTAAGG-3′ (Vrain, 1993) were used. The primers 5′-GGTCAATGTTCAGAAATTTGTGG-3′ and 5′-TACCTTTGACCAATCACGCT-3′ were used to amplified intergenic region between the cytochrome oxidase subunit II (COII)-16S rRNA of mitochondrial DNA (mtDNA) region (Powers and Harris, 1993). Accession numbers for all of the sequences of *M. aegracyperi* n. sp. have been submitted to the GenBank as follows: 18S: MN037410, ITS: MN044616, 28S: MN047211, COII: MN544409, and HSP90: MN544410.

## Results

Systematics

**Figure 1:**
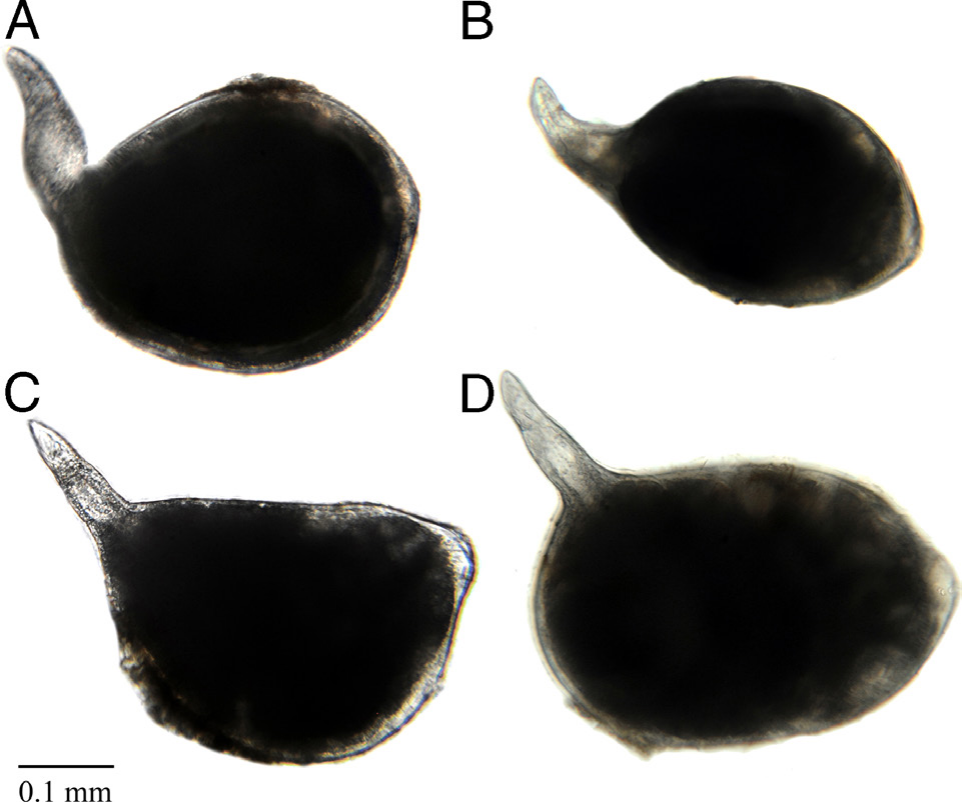
Females of *Meloidogyne aegracyperi* n. sp.; (A-D) Light micrographs of whole specimens showing the typical shape of the body and the posterior protuberance containing the perineal pattern (scale bar=0.1 mm).

**Figure 2:**
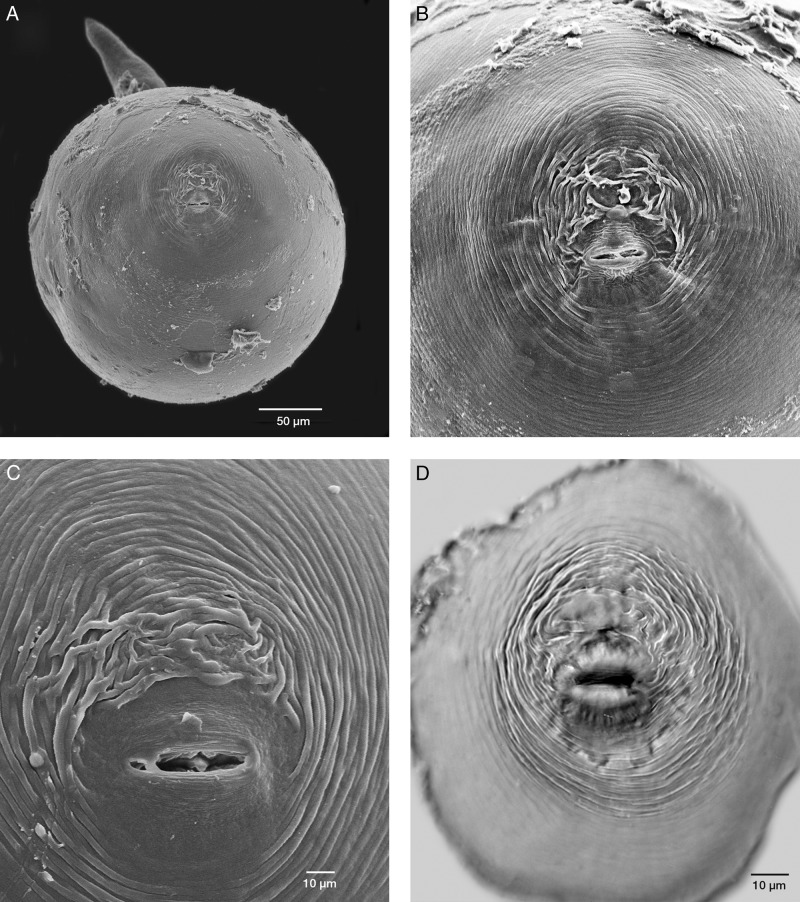
Scanning electron (SEM) and light micrographs of females of *Meloidogyne aegracyperi* n. sp.; (A) SEM of a whole female showing the location of the perineal pattern; (B) SEM of a perineal pattern situated on a protuberance of the posterior end of the body (magnification same as Fig. D); (C) SEM close-up of a perineal pattern; (D) Light micrograph showing the protuberance of the posterior end of the body containing the perineal pattern as seen in Fig. B.

**Figure 3:**
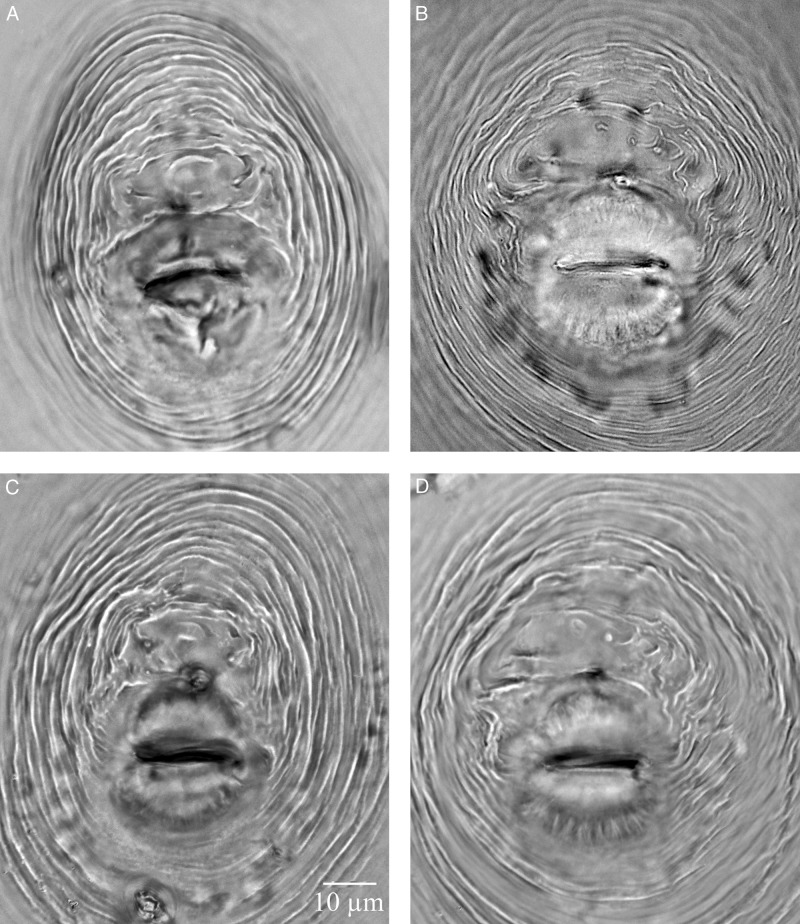
Light micrographs of perineal patterns of females of *Meloidogyne aegracyperi* n. sp.

**Figure 4:**
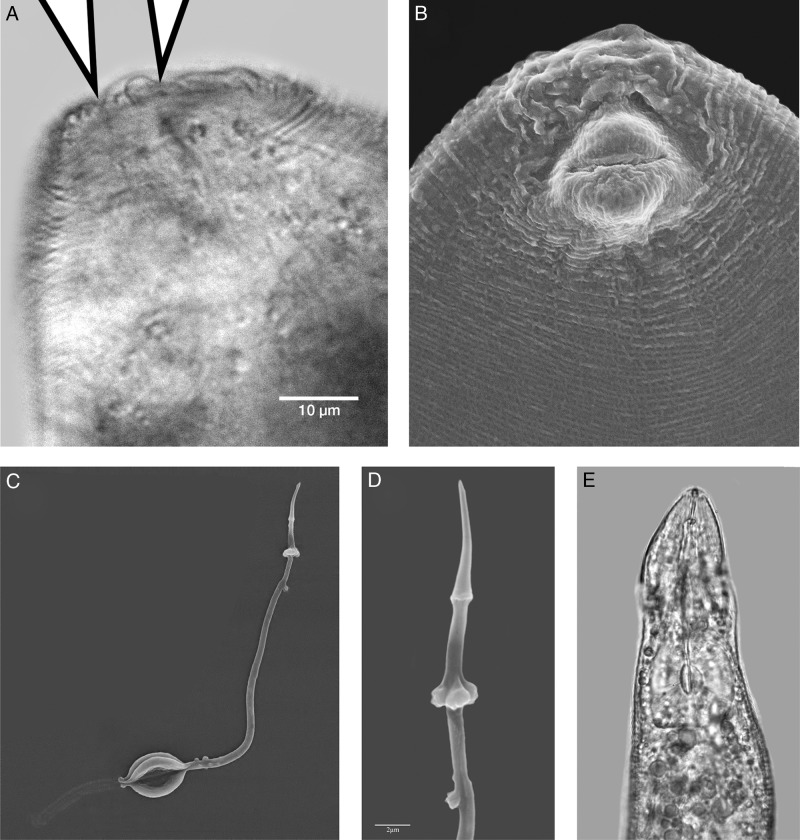
Light (LM) and scanning electron micrographs (SEM) of females of *Meloidogyne aegracyperi* n. sp.; (A) LM of posterior protuberance containing the perineal pattern with the anus and vulva marked by arrows; (B) SEM of posterior protuberance showing the swollen vulval lips and tail terminus; (C) SEM of the extracted cuticular lining of the esophagus with the stylet and showing the triradiate plates of the metacorpus and the vesicles in the lumen lining that are usually contained in the anterior region of the metacorpus; (D) SEM of an extracted stylet showing the angular edges on the knobs and the long dorsal esophageal gland orifice; (E) LM of the anterior end of the female showing the stylet and metacorpus.

**Figure 5:**
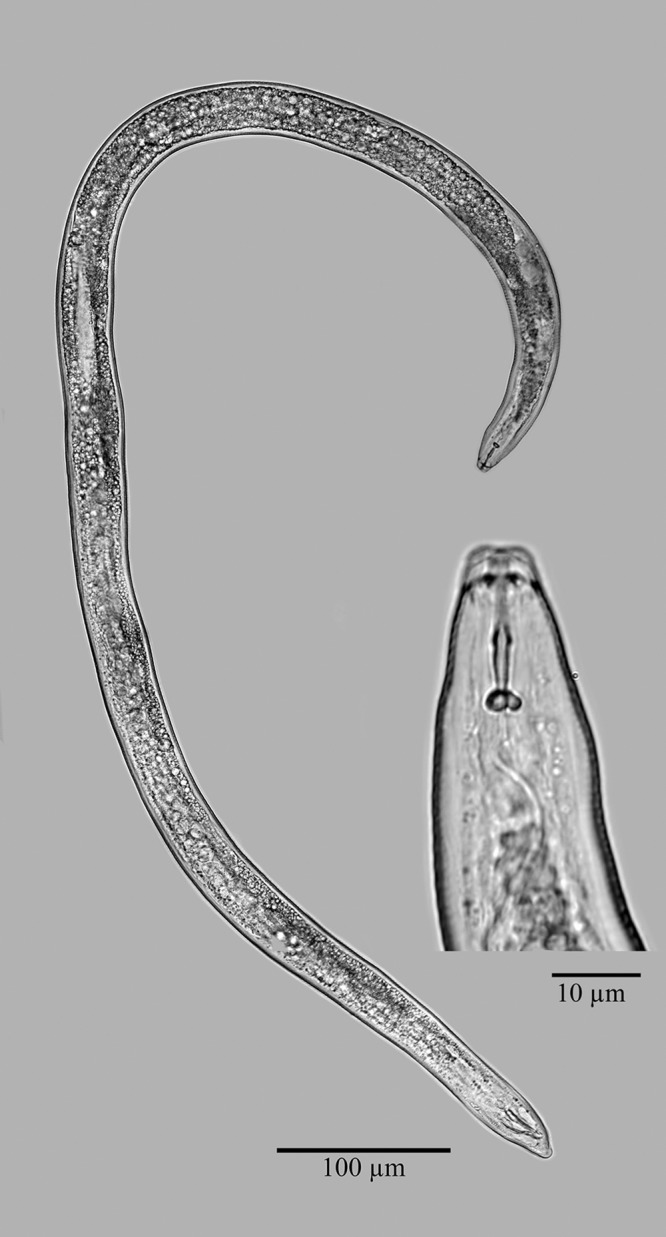
Light micrographs of a whole male specimen with an enlarged view of the anterior end of *Meloidogyne aegracyperi* n. sp.

**Figure 6:**
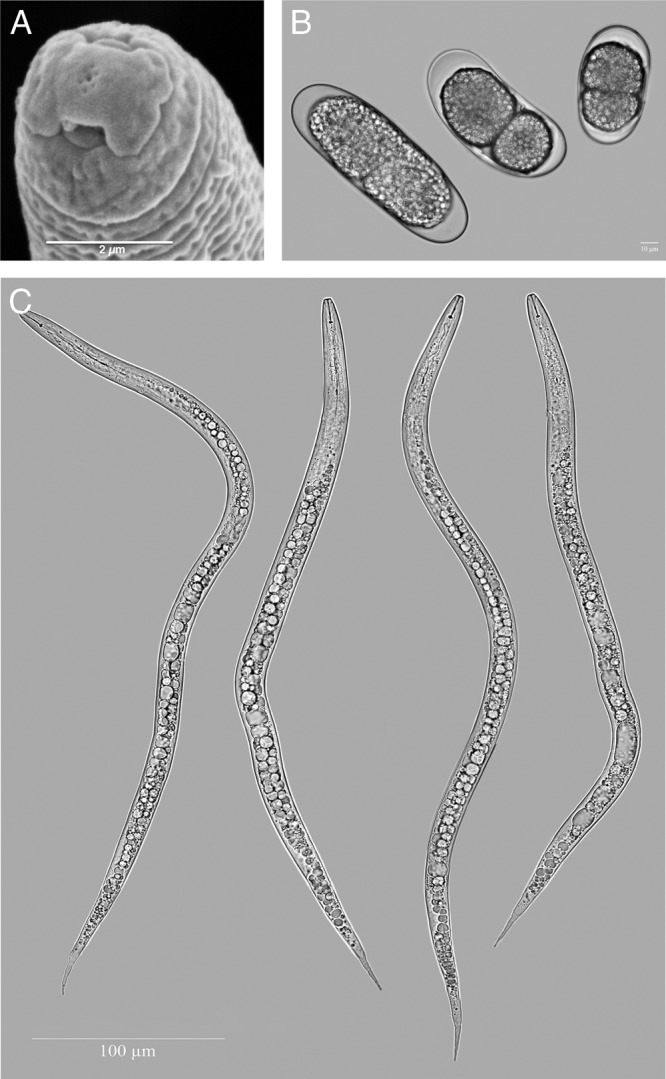
(A) Scanning electron micrograph of the anterior end of a second-stage juvenile of *Meloidogne aegracyperi* n. sp. (B) Light micrographs (LM) of eggs of *M. aegracyperi* n. sp. showing the variation in size for three eggs in the two-cell stage. Light micrographs of second-stage juveniles of *M. aegracyperi* n. sp.

**Figure 7:**
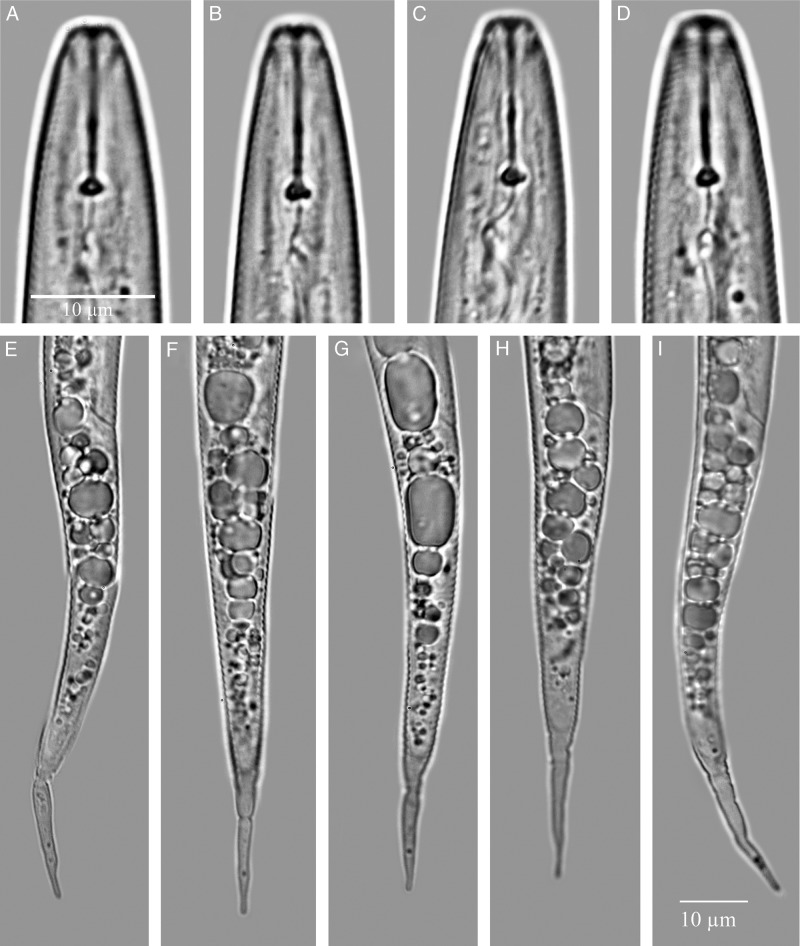
Light micrographs of the anterior ends and tails of second-stage juveniles of *Meloidogyne aegracyperi* n. sp.

*Meloidogyne aegracyperi* n. sp.

(Figs. 1-7; Table 2)

**Table 2.** Measurements and ratios of *Meloidogyne aegracyperi* n. sp. females, males, and second-stage juveniles.

	Female		Male	Second-stage Juv.
Character	Holotype	Paratypes	Paratypes	Paratypes
N	–	30	2	30
L	360	373 ± 44	1124 ± 10.5	426 ± 24.7
	–	(310–460)	(1113–1134)	(388–484)
Body diam.	292	306 ± 46	30.9 ± 1.4	15 ± 0.9
	–	(210–420)	(29.5–32.3)	(13.6–17.4)
Neck length	148	153 ± 27	–	–
	–	(100–210)	–	–
Stylet length	12	12 ± 0	15.6 ± 1.0	10.9 ± 0.4
	–	(12–12)	(14.6–16.5)	(10.1–11.8)
Stylet knob height	1.5	1.5 ± 0.2	2.3 ± 0.2	1.4 ± 0.2
	–	(1.2–1.9)	(2–2.5)	(1.1–1.9)
Stylet knob width	2.4	2.6 ± 0.3	4.1 ± 0.1	2.1 ± 0.2
	–	(2–3)	(4–4.2)	(1.7–2.5)
DGO	4.8	4.8 ± 0.6	3.1 ± 0.1	3.7 ± 0.5
	–	(4–6.1)	(3.0–3.3)	(2.7–4.8)
Stylet tip to metacorpus center	60.1	63.8 ± 2.2	–	–
	–	(59–67)	–	–
Interphasmidial distance	–	18 ± 2.7	–	–
	–	(14.2–24.4)	–	–
Vulva length	–	21 ± 2.9	–	–
	–	(13.5–26)	–	–
Vulva-anus distance	–	15.7 ± 2.3	–	–
	–	(10.9–21)	–	–
Ant. end to excretory pore	–	–	94.7 ± 1.6	68.5 ± 7.9
	–	–	(93.1–96.3)	(52–80.4)
Tail length	–	–	12.7 ± 1.9	73.1 ± 4.9
	–	–	(10.8–14.5)	(63.6–88.7)
Body width at anus	–	–	–	11 ± 0.6
	–	–	–	(10.2–11.9)
a	1.3	1.2 ± 0.2	–	28.4 ± 2.8
	–	(0.8–1.7)	–	(23.6–34.6)
c	–	–	–	5.8 ± 0.4
	–	–	–	(5.1–6.5)
Spicule length	–	–	23.6 ± 1.8	–
	–	–	(21.8–25.4)	–
Hyaline tail terminus	–	–	–	22 ± 2.0
	–	–	–	(18.5–26.6)

### Description

#### Female

Mature females with their egg masses are usually contained completely inside galled root tissues. They are very small (373 µm long) and pearly white. Their body shape is unique from many other species because the neck is often at a 90 to 130° angle to the protruding posterior end that contains the perineal pattern. Lip region low, cephalic framework weakly developed, with one head annule. The cone of the stylet slightly curved dorsally, posterior edges of the knobs angular, and tapering onto the shaft. The distance of the dorsal esophageal gland orifice (DGO) to the base of the stylet relatively long (4–6 µm). Excretory pore level, with the base of the stylet, is present. The lining of the metacorpus triradiate, with the posterior and anterior portions rounded. Numerous (3–10) small vesicles present in the anterior metacorpus. Two, small, rounded esophago-intestinal cells at the base of the metacorpus, followed by a large nucleated dorsal esophageal gland lobe with two smaller nucleated subventral esophageal gland cells. The didelphic ovary is typical for the genus. Six, large rectal gland cells connect to the rectum and produce the gelatinous matrix forming the egg mass. The perineal pattern is raised on a protuberance at the posterior end of the body. It contains a rounded dorsal arch with a tail terminal area that is usually smooth, but may be marked with thick lines and many horizontal, rope-like striae. Phasmids are typical for the genus. The vulval lips are usually flattened, but they may be rounded and slightly protruding. Smooth, regular striae surround the vulva and tail terminal area and give the appearance of a dorso-ventrally elongated oval pattern.

#### Male

The characteristics are as follows: anterior end tapering, labial disc slightly concave around the stoma, one distinct head annule, cephalic framework slight, stylet shaft tapering posteriorly. Body twisting 90° throughout its length. Stylet knobs rounded and set-off from the shaft. The distance of the DGO to the base of the stylet is long (3–3.3 µm). Esophageal glands overlapping the intestine ventrally. Four lines in the lateral field. Paired spicules with gubernaculum are typical for the genus. Tail tip slightly set-off from the remainder of the body.

#### Second-stage juvenile

It has a body with a very long tail and tail terminus. Cephalic framework is weak, stylet is small, with a constriction near the junction of the shaft and knobs. In SEM, the head has a slit-like oral opening placed on the rounded labial disc and surrounded by six small pit-like openings of the inner labial sensilla. Small depressions in the cuticle on the dorsal and ventral lip pairs mark the outer labial sensilla. Rounded knobs, tapering onto the shaft, are present. The distance of the DGO to the base of the stylet is long (3–5 µm). Esophageal glands overlap the intestine ventrally. Four lines in the lateral field are present. The tail is very long (64–89 µm) and the hyaline portion of the tail is very narrow, making the tail finely pointed. Phasmids are located midway between anus and tail tip.

#### Egg

Eggs are typical in shape for the genus and vary in length [91.6 ± 2.3 (85.2–99.8 µm)] and width [39.7 ± 0.1 (37.1–48.1 µm)], having a L/W ratio of [2.3 ± 0.1 (2.1–2.6). In one single mass of eggs, one egg was smaller than usual (69 × 43 µm), one was normal (95 × 42 µm), and one was large (125 × 43 µm), even though all were in a two-cell stage.

#### DNA sequence-based identification

DNA sequences were manually edited to remove low quality sequence and PCR priming regions prior to making assemblies for both the 18S rRNA and *Hsp90* loci. Ambiguity-free assemblies with an average length of 1,055 bp and covering ~2/3 of the 18S rRNA gene were generated for 12 of the 18S rRNA amplicons while assemblies with an average length of 754 bp were generated for 21 of the *Hsp90* amplicons. Consensus sequences from each assembly were used to construct multiple sequence alignments. No polymorphisms were detected in either the 18S rRNA or *Hsp90* multiple sequence alignments suggesting that all nematodes in the sample were from a clonal population. The 18S rRNA sequence spanned nt positions 603 to 1,797 relative to the 18S rRNA gene sequence reported for *M. arenaria* (Neal, 1889) Chitwood, 1949 (Genbank accession no. U42342). A BLAST search showed sequences from *M. graminicola* Golden and Birchfield, 1965 (Genbank accession no. LS974433) and *M. naasi* Franklin, 1965 (Genbank accession no. KP901048) were among the highest scoring matches to the 18S rRNA sequence from the sample specimens (99.89% identity over 100% of the sequence for each). The 786 nt consensus sequence obtained from *M. aegracyperi* n. sp. *Hsp90* spanned positions 75 to 864 relative to the *Hsp90* gene from *M. naasi* (Genbank accession no. KC262251). BLAST searches showed more divergence in the *Hsp90* gene than was seen in the 18S rRNA gene with the highest scoring matches being *M. naasi*, *M. minor* Karrsen, Bolk, van den Beld, Kox, Korthals, Molendijk, Zijlstra, van Hood, and Cook, 2004, and *M. fallax* Karssen, 1996 which were 89, 85, and 84% identical to *M. aegracyperi* n. sp., respectively.

**Figure 8:**
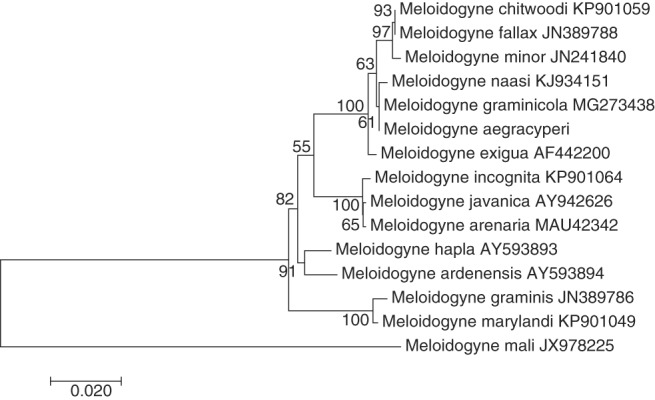
Maximum likelihood phylogenetic analysis of evolutionary relationships of closely related *Meloidogyne* species including NSRKN (*Meloidogyne aegracyperi* n. sp.) and comparison 18S rRNA sequences. The tree with the highest log likelihood (−2,286.9884) is shown. The bootstrap support values (500 bootstraps) are shown at nodes next to the branches. Branch lengths are proportional to the distances. Genbank accession numbers follow each species.

**Figure 9:**
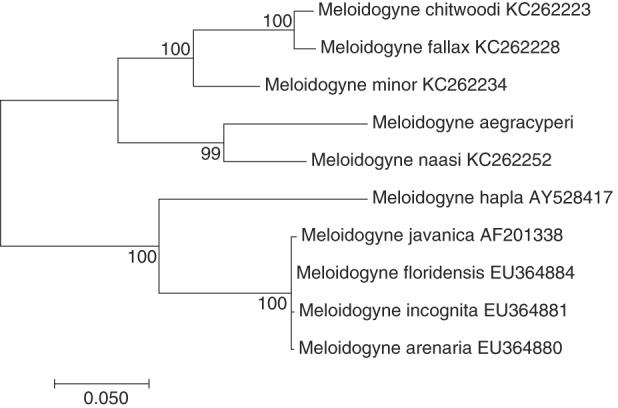
Maximum likelihood phylogenetic analysis of evolutionary relationships of closely related *Meloidogyne* species including NSRKN (*Meloidogyne aegracyperi* n. sp.) and comparison *Hsp90* sequences. The tree with the highest log likelihood (−2,926.7701) is shown. The bootstrap support values (500 bootstraps) are shown next to the branches. Branch lengths are proportional to the distances. Genbank accession numbers follow each species.

Maximum likelihood trees comparing *M. aegracyperi* n. sp. sequence to comparison sequences from GenBank were created for each gene using the maximum likelihood method based on the Tamura 3-parameter model (Tamura, 1992). Initial trees for the heuristic search were obtained automatically by applying Neighbor-Join and BioNJ algorithms to a matrix of pairwise distances estimated using the maximum composite likelihood (MCL) approach, and then selecting the topology with the highest log likelihood value. Evolutionary analyses were conducted in MEGA6 (Tamura et al., 2013). Both trees were robust with most branches, including *M. aegracyperi* n. sp. containing branches, having bootstrap support values well over 50%. Both trees also displayed similar topology with *M. aegracyperi* n. sp. sequences residing on an independent branch in between a branch containing *M. naasi* and/or *M. graminicola*, and a second branch containing sequences from *M. fallax*, *M. chitwoodi*, Golden, O'Bannon, Santo, and Finley, 1980 and *M. minor* (18S rRNA tree in Fig. 8, *Hsp90* tree in Fig. 9). While the 18S rRNA tree has more detail owing to more comparison sequences being available in Genbank, the *Hsp90* tree showed higher bootstrap support for all branches owing to more variability in this gene, with the majority of the variability coming from intron regions (data not shown).

**Figure 10:**
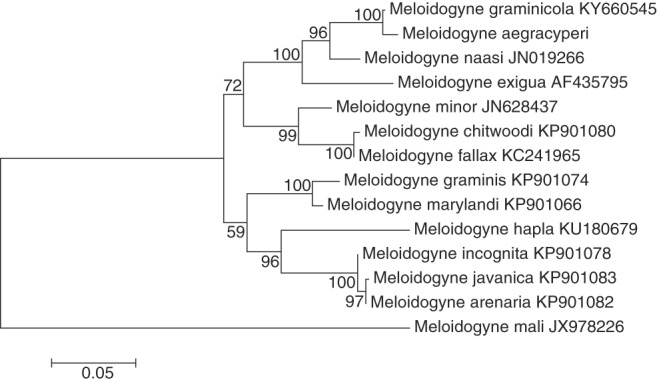
Maximum likelihood phylogenetic analysis of evolutionary relationships of closely related *Meloidogyne* species including *Meloidogyne aegracyperi* n. sp. and comparison of the alignment of the D2-D3 (28S rRNA) sequences. The tree with the highest log likelihood is shown. The bootstrap support values (500 bootstraps) are shown at nodes next to the branches. Branch lengths are proportional to the distances. Genbank accession numbers follow each species.

**Figure 11:**
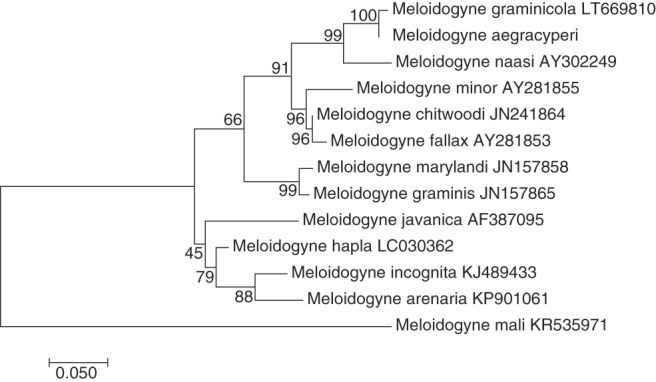
Maximum likelihood phylogenetic analysis of evolutionary relationships of closely related *Meloidogyne* species including *Meloidogyne aegracyperi* n. sp. and comparison of the alignment of the ITS sequences. The tree with the highest log likelihood is shown. The bootstrap support values (500 bootstraps) are shown at nodes next to the branches. Branch lengths are proportional to the distances. Genbank accession numbers follow each species.

**Figure 12:**
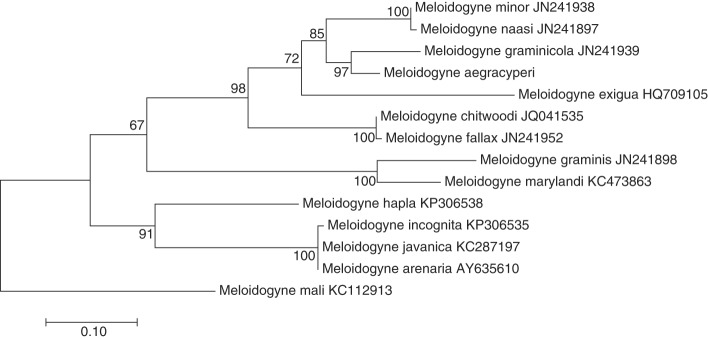
Maximum likelihood phylogenetic analysis of evolutionary relationships of closely related *Meloidogyne* species including *Meloidogyne aegracyperi* n. sp. and comparison of the alignment of the COII-16S rRNA sequences. The tree with the highest log likelihood is shown. The bootstrap support values (500 bootstraps) are shown at nodes next to the branches. Branch lengths are proportional to the distances. Genbank accession numbers follow each species.

The new species was also molecularly characterized using the D2-D3 fragment of the 28S rRNA, the ITS region of the rRNA, and the COII-16S rRNA region. In this case, only a single amplicon was sequenced for each of these *loci*. The D2-D3 region of 28S rRNA gene yielded an amplicon of 766 bp, including the primer sequences. Blast searches showed as top hit species several isolates of *Meloidogyne graminicola* (Genbank accession no. KY660545.1) and *M. naasi* (Genbank accession no. JN019266.1), with 99.13% and 94.96 identity, respectively. In the case of the ITS region, an amplicon of 634 bp was obtained containing both partial regions of the 18S and 28S rRNA. Similarly, different isolates of *M. graminicola* (Genbank accession no. LT669810) and *M. naasi* (Genbank accession no. AY302249) were among the top Blast hits, showing 93.45% and 86% identity against *M. aegracyperi* full amplicon, respectively. For the COII-16S rRNA region, several isolates of *M. graminicola* (Genbank accession no. MH033621) were among the top Blast hits, showing up to 90.99% identity against *M. aegracyperi* sequence. All generated trees generated for the D2-D3 and the ITS rRNA regions, and the COII-16S mitochondrial region, displayed similar topology with *M. aegracyperi* n. sp. sequences residing on an independent clade together with *M. graminicola* and *M. naasi*, and separated from other *Meloidogyne* species (Figs. 10–12).

Taken together, the congruent topologies obtained for all the tested molecular *loci* phylogenies suggest that *M. aegracyperi* n. sp. is a unique *Meloidogyne* species whose closest relatives are *M. graminicola*, *M. naasi, M. fallax*, *M. chitwoodi*, and *M. minor.*

#### Type host and locality

Lower Rio Grande Valley, Dona Ana County, New Mexico onion field on purple nutsedge (Rincon/Hatch Hwy 185, onion field, N32 39.431 W107 07.801).

#### Type material

The original population was derived from the type locality and host. Holotype female and 6 females and 10 second-stage juvenile paratypes isolated from a single egg mass and maintained on purple nutsedge in a greenhouse were deposited in the USDA Nematode Collection (USDANC), Beltsville, Maryland. Paratypes (3 females and 10 J2) were deposited in the Canadian National Collection of Nematodes, Ottawa, Canada.

#### Differential diagnosis

The most important measurements of females, males, and second-stage juveniles of *M. aegarcyperi* n. sp. are compared with those of the most closely related species, *M*. *naasi, M. fallax, M. minor*, *M. chitwoodi*, and *M. graminicola*, in Table 3. *Meloidogyne aegracyperi* n. sp. is characterized by the small female (373 µm long × 306 µm in diameter) with a perineal pattern that occurs on a posterior protuberance that is at a 90 to 130° angle with the neck. In *M. naasi*, the female is larger (557 µm long × 330 µm in diameter) (Franklin, 1965) similar to that of *M. graminicola* (573 µm long × 419 µm in diameter) (Golden and Birchfield, 1965). The perineal pattern of *M*. *naasi* is often on a protuberance, and it is rounded to oval-shaped with striae that completely encircle the tail terminus, anus, and vulva; the tail terminus is usually smooth, but may contain rope-like striae that are parallel with the vulva (Franklin, 1965). The perineal pattern of *M. graminicola* is typically flat (Golden and Birchfield, 1965). The smooth region around the tail of *M. aegracyperi* n. sp. makes it different from that of *M. naasi* which usually contains rope-like striae that are perpendicular to the vulva. The edges of the stylet knobs are angular, the stylet is short (12 µm), and the DGO is long (3.6–6.1 µm) in *M. aegracyperi* n. sp., whereas in *M. naasi* the edges of the stylet are smooth, the stylet is longer (13 µm), and the DGO is shorter (2–4 µm) (Franklin, 1965). In *M. graminicola*, the stylet is shorter (11 µm) and the DGO is similar to that of *M. naasi* (3–4 µm) (Golden and Birchfield, 1965). Males are very rare in *M. aegracyperi* n. sp. which may be a useful diagnostic character since males are common in *M. naasi* and *M. graminicola.* The second-stage juvenile resembles that of *M. naasi*; however, the body is slightly shorter (426 vs 435 µm), the stylet is much shorter (10.8 vs 14 µm), and the DGO is much longer (3.7 vs 2.4 µm). Likewise, the tail is longer (73.1 vs 70 µm) and the hyaline portion of the tail is also longer than that of *M. naasi* (22 vs 17.5 µm) (Franklin, 1965). The second-stage juvenile of *M. graminicola* is longer (441 µm), the stylet is shorter (11.4 µm), and the DGO is more like *M. naasi* in length (3.2 µm) (Golden and Birchfield, 1965). In *M. aegracyperi* n. sp., the tail and its terminus are longer (73.1 µm and 22 µm, respectively), whereas both measurements are shorter, but similar to each other in *M. naasi* (70 µm and 17.5 µm, respectively) (Franklin, 1965) and *M. graminicola* (70.9 µm and 17.9 µm, respectively) (Golden and Birchfield, 1965).

**Table 3.** Comparisons of key measurements of females, second-stage juveniles, and males of *Meloidogyne aegarcyperi* n. sp. with its closest-related species: *M. graminicola* Golden and Birchfield, 1965; M. *naasi* Franklin, 1965; *M. fallax* Karrsen, 1996; *M. minor Karssen*, Bolk, van den Beld, Kox, Korthals, Molendijk, Zijlstra, van Hood, and Cook, 2004; and *M. chitwoodi* Golden, O'Bannon, Santo, and Finley, 1980.

*Female*					
Species	Length	Width	Stylet	DGO	Vulva
*M. chitwoodi* (*n*=60)^a^	(430–750) 591	(344–518) 422	(11.2–12.5) 11.9	(3.4–5.5) 4.2	
*M. fallax* (*n*=30)^b^	(404–720) 491	(256–464) 362	(13.9–15.2) 14.5	(3.8–6.3) 4.3	(20.2–28.4) 24.7
*M. minor* (*n*=25)^c^	(416–608) 526	(240–464) 339	(12.6–15.2) 14.2	(3.2–6.3) 4.1	(22.8–29.1) 25.8
*M. naasi* (*n*=25)^d^	(455–705) 557	(227–398) 330	(11–15) 13	(2–4) 3	(17–25) 22
*M. graminicola* (*n*=20)^e^	(455–765) 573	(275–520) 419	(10.6–11.2) 11.1	(2.8–3.9) 3.2	
*M. aegracyperi* n. sp. (*n*=30)	(310–460) 373	(210–420) 306	(12–12) 12	(4–6.1) 4.8	(13.5–26) 21
*Juvenile*					
Species	Length	Stylet	DGO	Tail	Terminus
*M. chitwoodi* (*n*=60)^a^	(336–417) 390	(9.0–10.3) 9.9	(2.6–3.9) 3.2	(39–47) 43	(8.6–13.8) 11
*M. fallax* (*n*=30)^b^	(381–435) 403	(10.1–11.4) 10.8	(3.2–3.8) 3.5	(46.1–55.6) 49.3	(12.2–15.8) 13.5
*M. minor* (*n*=25)^c^	(310–416) 377	(7.6–10.1) 9.2	(3.2–4.4) 3.8	(58.1–77.1) 54	(12.0–22.1) 16.1
*M. naasi* (*n*=25)^d^	(418–465) 435	(13–15) 14	(2–3) 2.4	(52–78) 70	17.5
*M. graminicola* (*n*=20)^e^	(415–484) 441	(11.2–12.3) 11.4	(2.8–3.4) 2.8	(67.0–76.0) 70.9	(14.0–21.2) 17.9
*M. aegracyperi* n. sp. (*n*=30)	(388–484) 426	(10.1–11.8) 10.8	(2.7–4.8) 3.7	(63.6–88.7) 73.1	(18.5–26.6) 22
*Male*					
Species	Stylet	DGO			
*M. chitwoo*di (n–30)^a^	(18.1–18.5)18.3	(2.2–3.4) 3			
*M. fallax* (*n*=30)^b^	(18.9–20.9) 19.6	(3.2–5.7) 4.4			
*M. minor* (*n*=25)^c^	(17.1–19.0) 17.8	(3.2–4.4) 3.8			
*M. naasi* (*n*=25)^d^	(16–19) 18	(2–4) 3			
*M. graminicola* (*n*=20)^e^	(16.2–17.4) 16.8	(2.8–3.9) 3.3			
*M. aegracyperi* n. sp. (*n*= 2)	(14.6–16.5) 15.6	(3.0–3.3) 3.1			

Length = maximum body length; Width = maximum body width; Stylet = maximum stylet length; DGO = length of the dorsal gland orifice to the base of the stylet knobs; Vulva = maximum width of the vulva; Tail = distance from the anus to the tail tip; Terminus = distance of the hyaline portion of the tail to the tip of the tail.

^a^Golden et al. (1980); ^b^ Karssen (1996); ^c^ Karssen et al. (2004); ^d^ Franklin (1965); ^e^Golden and Birchfield (1965).

Comparisons of males of these three species reveal that the DGO is very similar (3–3.1 µm), but the length of the stylet is quite dissimilar [*M. aegracyperi* n. sp.=15.6 µm; *M. naasi*=18 µm (Franklin, 1965); and *M. graminicola* = 16.8 µm (Golden and Birchfield, 1965)].

The host ranges of *M. aegracyperi* n. sp., *M. naasi*, and *M. graminicola* are very different (Table 4). The common hosts of *M. aegracyperi* n. sp., and *M. naasi* as reported by Franklin (1965) are winter rye, wheat, and barley. However, Radewald et al. (1970) listed alfalfa and cotton as hosts of *M. naasi*, but Allen et al. (1970) considered them to be non-hosts. Cotton, alfalfa, and sorghum were non-hosts of *M. aegracyperi* n. sp., unlike the ambiguous results reported for *M. naasi*. The questions remain: Were Radewald and Allen working with the same species, or does *M. naasi* occurs as several different host races (Allen et al., 1970; Radewald et al., 1970; Michell et al., 1973)?

**Table 4.**
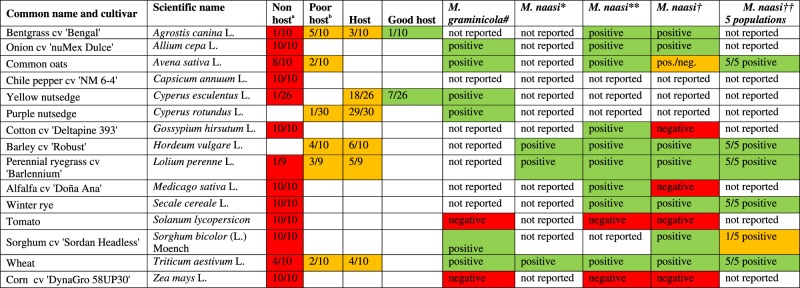
Hosts and non-host plants of *Meloidogyne aegracyperi* n. sp. compared to eight populations of its closest-related species: *M. graminicola* Golden and Birchfield, 1965 (Minton et al., 1987; Soomro and Hague, 1992a,b); and *M. naasi* Franklin, 1965, reported by Franklin (1965) from the type population in England; Radewald et al. (1970) and Allen et al. (1970) from California; and Michell et al. (1973) from England, California, Illinois, Kansas, and Kentucky.

August 13, 2008 - planted and inoculated with 5,000 per pot. *Franklin, 1965;**Radewald et al., 1970; †Allen et al., 1970; and ‡Michell et al., 1973 tested populations from England, California, Illinois, Kansas, and Kentucky; ^a^Proportion of 10 plants that exhibited host characteristics; ^b^poor host = RF (ratio of eggs recovered/inoculum level) > 0 but < 1; host = RF > 1 but < 10; good host = RF > 10; red = non or poor host, yellow = non and good host, and green = good host.

The common hosts of *M. aegracyperi* n. sp. and *M. graminicola* include yellow and purple nutsedge and wheat; however, *M. graminicola* parasitizes onion, common oats, and sorghum (Minton et al., 1987), but *M. aegracyperi* n. sp. cannot.

## Discussion

*Meloidogyne aegracyperi* n. sp. is morphologically similar to *M*. *naasi*, but it can be distinguished as a unique species based on features of the female, male, and second-stage juvenile. Superficially, the gross morphology of the second-stage juvenile and similar appearance of the perineal pattern of the female could cause a wrong identification of *M. aegracyperi* n. sp. as *M. naasi*. However, measurements of the stylets, DGO, body length, tail length, and hyaline tail terminus easily separate these two species. *Meloidogyne aegracyperi* n. sp. appear to be closely related phylogenetically to *M. graminicola* and *M. naasi* according to the trees that were drawn based on similarities of DNA sequences of the 18s rRNA, D2-D3 region of rRNA, ITS region, COII-16S, and *Hsp90* genes. Likewise, their host ranges feature some common species including oats, barley, bentgrass, and wheat (Franklin, 1965; Allen et al., 1970; Radewald et al., 1970), but host status of alfalfa, cotton, and sorghum easily separate *M. naasi* from *M. aegracyperi* n. sp.
